# The Primary Prevention of Poststroke Epilepsy in Patients With Middle Cerebral Artery Infarct: Protocol for a Randomized Controlled Trial

**DOI:** 10.2196/49412

**Published:** 2023-11-24

**Authors:** Yu-Shiue Chen, Pi-Shan Sung, Ming-Chi Lai, Chin-Wei Huang

**Affiliations:** 1 Department of Neurology National Cheng Kung University Hospital, College of Medicine National Cheng Kung University Tainan Taiwan; 2 Department of Pediatrics Chi-Mei Medical Center Tainan Taiwan

**Keywords:** poststroke epilepsy, middle cerebral artery infarct, seizure, randomized control trial, epilepsy, stroke, stroke survivor, prognosis, mortality, drug therapy, development, seizure severity, efficacy, heart attack, angina, cardiovascular, cardiology, cardiologist

## Abstract

**Background:**

Poststroke epilepsy poses a significant clinical challenge for individuals recovering from strokes, leading to a less favorable long-term outlook and increased mortality rates. Existing studies have primarily concentrated on administering antiseizure or anticonvulsant treatments only after the onset of late-onset seizures, without intervening during the epileptogenesis phase following a stroke.

**Objective:**

This research protocol is designed to conduct a randomized controlled trial to assess whether the early, preventive introduction of low-dose antiepileptic drug therapy (levetiracetam [LEV] or perampanel [PER]) in patients who have experienced middle cerebral artery (MCA) infarction can reduce the risk of developing poststroke epilepsy (primary prevention).

**Methods:**

Participants with MCA infarction, either with or without reperfusion treatments, will be recruited and promptly receive preventive intervention within 72 hours of the stroke occurrence. These participants will be randomly assigned to receive either PER (4 mg per day), LEV (1000 mg per day), or a placebo that matches the active drugs. This treatment will continue for 12 weeks after allocation. Brain magnetic resonance imaging will be used to confirm the presence of MCA territory infarction, and an electroencephalography will be used to ensure the absence of epileptiform discharges or electrographic seizures at the time of the stroke. All participants will undergo follow-up assessments for 72 weeks after allocation.

**Results:**

The primary outcome under evaluation will be the incidence of poststroke epilepsy in the 3 groups following the 18-month study period. Secondary outcomes will encompass the time to the occurrence of the first seizure, the severity of seizures, any treatment-related adverse events, and the modified Rankin scale score at 3 and 18 months. Exploratory outcomes will involve comparing the effectiveness and safety of PER and LEV.

**Conclusions:**

We anticipate that the intervention groups will experience a lower incidence and reduced severity of poststroke epilepsy compared to the control group after 18 months. We aim to establish evidence supporting the potential preventive effects of LEV and PER on poststroke seizures and epilepsy in patients with MCA infarction, as well as to explore the antiepileptogenic potential of both LEV and PER in patients with major ischemic strokes.

**Trial Registration:**

ClinicalTrials.gov NCT04858841; https://clinicaltrials.gov/study/NCT04858841

**International Registered Report Identifier (IRRID):**

DERR1-10.2196/49412

## Introduction

### Background

Advances in stroke treatment have caused a considerable reduction in stroke mortality; however, the number of stroke survivors living with disability has increased significantly. Seizures and epilepsy related to ischemic stroke are common, and poststroke epilepsy is a significant clinical problem for stroke survivors. Stroke is the most common cause of epilepsy in older adults and for patients older than 65 years of age, and poststroke epilepsy accounts for 30%-50% of new-onset seizures [1]. The incidence of early seizures (occurring within the first 1-2 weeks of stroke) is 2.4%-5.4%, and the risk of poststroke late seizures (seizures occurring later than after 14 days of stroke) is approximately 7%-18% [1,2]. Late-onset poststroke epilepsy has a higher recurrence rate of up to 71.5% within 10 years after stroke [3]. Our previous study demonstrated that seizures during stroke increase overall morbidity and mortality [4]. The stroke severity, location (for example, middle cerebral artery [MCA] infarcts in our study), and the type of pathological changes, genetic factors, and pre- and postinjury exposure to nongenetic factors influence the susceptibility of patients with ischemic stroke [5,6]. The standardized morbidity rate of developing epilepsy is highest during the first year after stroke. In addition, higher National Institutes of Health Stroke Scale (NIHSS) score [7], cortical involvement, younger age, and central nervous system morbidities are associated with higher risk of poststroke epilepsy [1,8].

The effects of thrombolysis and thrombectomy on long-term poststroke epilepsy remain unclear. A retrospective evaluation of patients with thrombolysis found that 4% developed poststroke seizures during hospitalization and that death during admission was significantly more likely in those with seizures compared to those without seizures [9]. Another study found that 5.5% of patients receiving endovascular therapy had seizures between stroke onset and 3-month follow-up, and early seizures independently predicted a significantly unfavorable outcome [10]. Regarding early postthrombectomy seizures, 2.4% of patients with ischemic stroke treated with thrombectomy developed at least 1 seizure within 7 days of stroke onset, and the occurrence of seizures was associated with higher 90-day mortality and poorer functional outcomes [11]. For patients with stroke, receiving reperfusion treatments was not associated with acute symptomatic poststroke seizures or poststroke epilepsy [7,12].

Ethnicity may be an important factor in the development of poststroke seizures and epilepsy. The prevalence of poststroke seizures in patients without atrial fibrillation is higher in Australia than in China [13]. In Taiwan, 1 study found that atrial fibrillation may cause ischemic stroke that subsequently causes seizure and atrial fibrillation is associated with a higher rate of subsequent seizure [14]. One-year poststroke epilepsy risk is higher in patients staying in the ICU or in patients with seizure at stroke admission, atrial fibrillation, or cognitive impairment [15]. Late poststroke seizure recurrence is more common in patients of young age (younger than 65 years of age), of male sex, with large lesion seizure, or with partial-onset seizure [16].

### Concept of Epileptogenesis

Antiepileptic drugs (AEDs) or antiseizure medications may prevent the recurrence of seizures but do not cure epileptogenesis. An epileptogenesis is the process by which the previously normal brain is functionally altered and biased towards the generation of abnormal electrical activity that induces chronic seizures [17]. It involves molecular, anatomical, or circuit-level alterations, such as cell death or dysregulation of an ion channel or neurotransmitter receptor and the impairment of intrinsic plasticity. How these different levels of alteration are regulated remains unclear. Studies have reported that the epigenetic factor chromodomain Y-like protein, a critical regulator of the initiation and maintenance of intrinsic neuroplasticity that works by regulating voltage-gated sodium channels [18], is involved, and epileptic neurons may also develop short- and long-term adaptive changes in sensitivity to gamma-aminobutyric acid (GABA)ergic neurotransmission [19], worsening excitatory or inhibitory imbalance and thus reducing the possibility of successful therapeutic approaches using traditional AEDs. Further, an increasing amount of research, including ours, suggests a major involvement of inflammation in epileptogenesis [20-22]. Seizure activity elicits the release of proinflammatory cytokines and activates immune responses.

We have studied medications with potential antiepileptogenesis effects in pilocarpine-induced seizure animal models, supporting the concept of antiepileptogenesis with intervention immediately following brain insult [21-23].

### Studies Regarding Primary Prevention

The clinical trials aimed at the prevention of chronic epilepsy have often produced negative results because they mainly evaluated downstream clinical manifestations instead of monitoring early, specific molecular epileptogenic events and applying early intervention. A 1990 study on primary prevention of posttraumatic seizures with early use of phenytoin [24] indicated that phenytoin exerts a beneficial effect by reducing seizures only during the first week after severe head injury.

American Heart Association guidelines indicate that prophylactic AEDs are not recommended for the prevention of poststroke seizure [25] due to the lack of randomized control trials and some reports indicating that prophylactic AED therapy may be associated with poorer outcomes [26-28]. In addition, valproic acid therapy was found to be ineffective compared with a placebo in preventing the occurrence of late-onset seizures in patients with spontaneous and nonaneurysmal intracerebral hemorrhage [29]. Nevertheless, the lack of success in prophylactic AED therapy trials may be related to the use of traditional AEDs.

### Medications With Antiepileptogenetic Potential

Numerous AEDs exist. Based on our clinical experience and laboratory research [21,22,30-33], we have identified 2 newer AEDs with antiepileptogenesis potential. Levetiracetam (LEV), which exhibits a distinct synaptic vesicle modulating mechanism, has exhibited the potential in ameliorating epileptogenesis [1,34,35]. Our earlier research indicates that it has a broad spectrum of modulating neuronal excitability [30]. It exhibits no clinically relevant drug-drug interactions [36]. Perampanel (PER), a standard AED with a distinct mechanism of selective noncompetitive antagonism of alpha-amino-3-hydroxy-5-methyl-4-isoxazolepropionic acid (AMPA)-type receptors, is also effective in treating a wide spectrum of epileptic seizures and has a favorable tolerability profile [37]. Because it blocks glutamate excitotoxicity, PER is theoretically capable of modulating epileptogenesis and has demonstrated potential in ameliorating epileptogenesis [32,38,39]. Thus, it is potentially justified to evaluate whether early prophylactic administration of LEV or PER in patients with acute major stroke hampers the development of epileptogenesis and later poststroke epilepsy.

## Methods

### Overview

We will conduct a prospective randomized, triple-blind, and placebo-controlled study. To calculate the appropriate sample size, we took into account various factors including the population size, margin of error, confidence level, expected variance, number of patients with MCA infarcts admitted to our medical center annually, and potential loss rate. We used G*Power software (Heinrich-Heine-Universität Düsseldorf) to perform these calculations. Specifically, we used an effect size ranging from 0.25 to 0.4, α error probability of .05, power of 0.5, 3 groups, 2 measurements, and a correlation of 0.05 between repeated measures. The resulting total sample size ranged from 51 to 120. Based on the potential number of patients admitted to our medical center and the expected loss rate, we estimated that enrolling 180 cases would be reasonable.

### Study Participants and Eligibility Criteria

We will recruit patients on the basis of the inclusion criteria of exhibiting acute MCA infarct and an NIHSS score of >8 (based on Diener Law) [40], confirmed using brain magnetic resonance imaging (MRI) studies during hospital admission within 72 hours of symptom onset. The exclusion criteria will be as follows: clinical history of major stroke; significant head injury; brain tumor; major psychiatric illness; progressive neurodegenerative disorder; central nervous system infection; epilepsy that may precipitate seizures; use of AEDs, including LEV or PER; being pregnant or lactating; history of inadequate medical compliance; or any other significant major systemic disease with safety concerns, as determined by a physician.

### Intervention

The participants will be divided into 3 groups: the control (placebo) group and the 2 experimental groups (LEV and PER). The patients, investigator, study assessors, and data analysts will remain blind to the treatment assignments from the time of randomization. Because we will conduct block randomization for group allocation, we anticipate that the percentage of patients receiving tissue plasminogen activator or intravenous thrombectomy will be similar in the 3 groups.

The major MCA infarctions typically require the early implantation of a nasogastric tube, and to account for the design of blinding, preventive drugs will be administered in powdered form. The study team works with pharmacists to ensure that medications for the experimental and control groups have similar color, fragrance, and taste. After being ground up, the drugs are packaged in identical medication bags, and a reminder label is affixed to the outer packaging to remind patients to take their medication regularly and for the appropriate duration.

**Figure 1:**
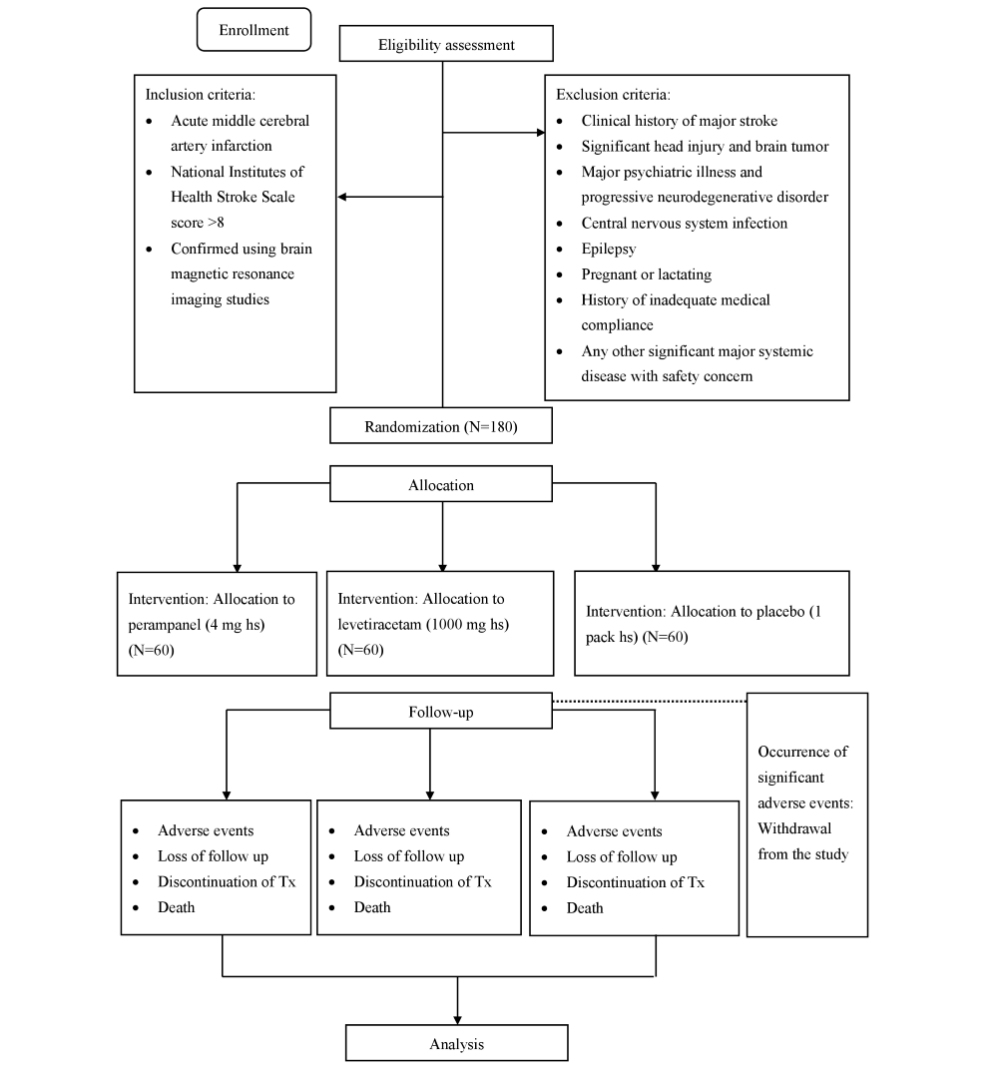
Randomized controlled trial flow diagram. Tx: Treatment.

The three groups are described as follows, and the study flowchart is shown in Figure 1: (1) For the control group, a placebo will be administered to patients, and we will maintain standard stroke care during hospital admission if no acute seizure occurs during this period. If an acute seizure occurs, unblinding will be performed, and we will administer AEDs not including LEV or PER; the patient will be maintained on these AEDs. (2) For the LEV group, we will prophylactically administer 1000 mg of LEV per day to patients from the first day of admission in addition to standard stroke treatment. The LEV treatment duration will be 3 months. (3) For the PER group, we will prophylactically administer 4 mg of PER per day to patients from the first day of admission in addition to standard stroke treatment. The PER treatment duration will be 3 months. (4) After the patient is discharged from the hospital, their respective medication will be administered orally for up to 3 months. (5) All patients will receive follow-up for 18 months, and the incidence of late-onset epilepsy will be documented. Seizure characteristics, including semiology, frequency, and severity, will be analyzed in the 3 groups. The relationship of seizure semiology to stroke-related localization will be verified. (6) For those who have acute seizures during hospital admission, unblinding will be performed, and either LEV or PER will be administered for treatment according to their group. The dosage will be adjusted according to real clinical needs. These patients will be treated as having epilepsy based on the 2016 definition of epilepsy [41] and be maintained on LEV or PER after hospital admission. The administration of LEV or PER to patients with acute seizures will still be considered prophylactic therapy because patients with acute seizures have a considerably higher risk of developing poststroke epilepsy. (7) Because up to 20% of patients with anterior circulation ischemic stroke may experience electrographic seizures during the acute stage of stroke without clinically overt seizures [42], we will arrange 1 routine electroencephalogram (EEG) at the acute stage for each patient. Prophylactic AED therapy will be administered according to the group the patient is allocated to if any electrographic seizure, including periodic lateralized epileptiform discharge (PLED), is identified in the EEG. (8) Unblinding will be implemented at 3 months, and the final analysis will be conducted at 18 months.

### Randomization and Blinding

**Figure 2:**
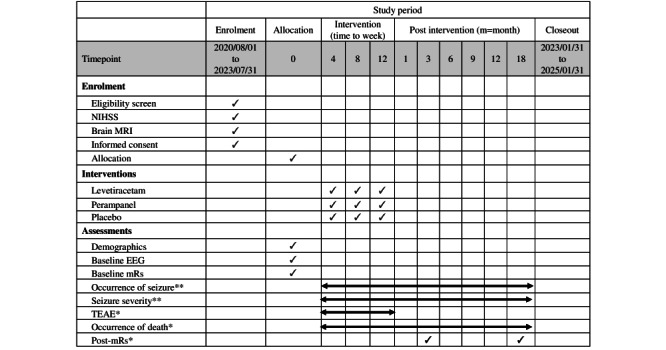
SPIRIT flow diagram: schedule of enrollment, interventions, and assessment procedures. EEG: electroencephalography; MRI: Magnetic Resonance Imaging; mRs: modified Rankin Scale; NIHSS: National Institute of Health Stroke Scale; SPIRIT: Standard Protocol Items: Recommendations for Interventional Trials; TEAE: treatment-emergent adverse event; **: primary outcome; *: secondary outcome.

Block randomization of eligible participants will be performed to form the LEV, PER, and placebo groups. Blinding of participants, clinicians, and data collectors will be performed so that these individuals do not have access to group assignment details (Figure 2). Before accepting the case, the pharmacist, who is a nonresearch team member, sets the blocks and determines the distribution of random numbers using a random number table. The pharmacist is responsible for conducting all phases of the research. Additionally, the 3 topic groups can be equally distributed among participants through random assignment.

### Procedure and Assessment

**Figure 3:**
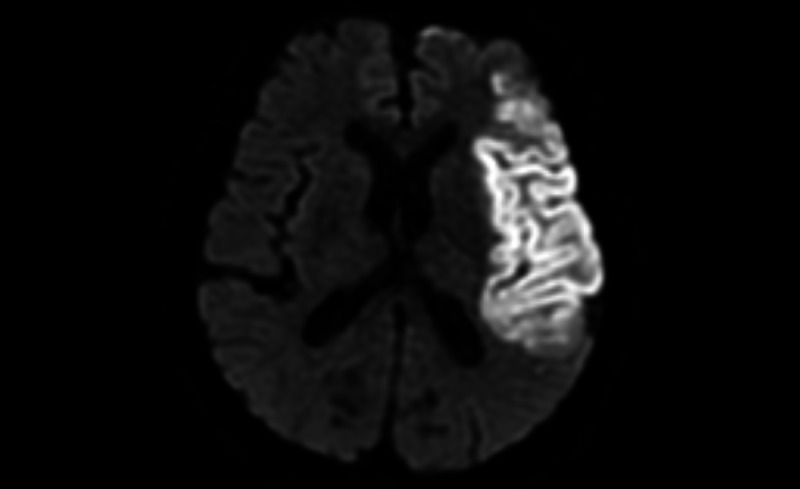
A typical case with middle cerebral artery infarct enrolled in this study.

We will document patients with initial NIHSS scores of >8 [40]. For reperfusion therapy, we will also use a modified Rankin scale for treatment evaluation [43]. For each participant, we will use brain MRI to confirm MCA territory infarction and exclude that of other territories and lacunar infarction. The MRI image depicting the typical case with an MCA infarct enrolled in this study is displayed in Figure 3. Baseline EEG during admission prior to AED administration will be arranged to exclude patients with early or concurrent clinical or electrographic seizures (Figure 2).

### Outcome Measures

#### Primary Outcome

The primary outcome measure will be the incidence of poststroke epilepsy and seizure severity at the end of the study period. Univariate ANOVA of the LEV, PER, and placebo groups will be performed. Intention-to-treat analysis will be performed. Participants will be followed up 1, 3, 6, 9, 12, and 18 months after stroke. If a seizure occurs during the prophylactic medication administration period, individual unblinding and adjustment of AEDs will be implemented. If a seizure occurs after the prophylactic medication administration period, ordinary medical care will be provided. We will record seizure type and duration after stroke onset at seizure occurrence **(**Table 1 and Figure 2).

**Table 1:** Summary of follow-up visits.

Visiting time	Within 3 days	3 months	6/9/12 months	18 months
NIHSS^a^	✓			
mRs^b^	✓			✓
MRI^c^	✓			
EEG^d^	✓			
Start of medication	✓			
TEAE^e^	✓	✓		
Occurrence of seizure	✓	✓	✓	✓
Occurrence of death	✓	✓	✓	✓

^a^NIHSS: National Institute of Health Stroke Scale.

^b^mRs: modified Rankin scale.

^c^MRI: magnetic resonance imaging.

^d^EEG: electroencephalograph.

^e^TEAE: treatment-emergent adverse event.

#### Secondary Outcomes

The secondary outcomes will include the duration until the occurrence of the first seizure, the severity of seizures, the safety and tolerance of the medication, and the overall stroke outcome. During the 18-month follow-up, we will record mortality, medication safety, and tolerability data at each visit. Stroke outcome will be measured using the modified Rankin scale at 3 and 18 months (Table 1 and Figure 2).

### Statistical Analysis

Continuous variables will be assessed using a 2-tailed *t* test or ANOVA, followed by the Fisher least significant difference test. When data are not normally distributed, analyses will be performed using the nonparametric ANOVA Kruskal-Wallis H test followed by Dunn multiple comparison test for nonrepeated measures and the Friedman test for repeated measures. ANOVA will be used where sessions or quadrants will be treated as a within-subject factor and groups as a between-subjects factor. Analyses will be performed using a chi-square test, Yates chi-square test, or Fisher exact test for nominal variables. Differences will be considered statistically significant at a *P* value <.05, and actual *P* value will be reported, unless *P*<.001.

### Ethical Considerations

This study received approval from the institutional review board (IRB) at National Cheng Kung University Hospital (A-BR-108-107). Each participant and their families were provided with a comprehensive verbal explanation of the study and were asked to provide informed consent, as authorized by the IRB. Participants completed their signatures on the consent forms to indicate their agreement to participate in the study.

The study data would be kept anonymous and deidentified to protect the participants’ privacy. Participants were not offered any form of compensation or reimbursement for their involvement in this human subjects’ research.

## Results

Participant recruitment began on August 1, 2020 in National Cheng Kung University Hospital. Intervention, postintervention, and follow-up observation are still ongoing for the participants. The study is projected to be completed by January 31, 2025.

## Discussion

### Principal Findings

Although reports have not supported the role of prophylactic AEDs in the prevention of poststroke seizure because of a lack of randomized control trials, a lack of detected early clinical biomarkers, and worse outcomes with traditional AEDs, we will document the potential prophylactic effect of LEV and PER on poststroke seizures and epilepsy in patients with MCA infarct in this randomized clinical trial and determine the antiepileptogenic potential of LEV and PER in patients with major ischemic stroke in terms of seizure frequency and severity. We will also evaluate the safety and tolerability of these prophylactic treatments. This study will influence both stroke and epilepsy care considerably, including by potentially reducing long-term care costs and enhancing the quality of life of stroke survivors.

In addition to LEV, previous studies, including our own, have identified PER as a new AED with potential antiepileptogenic properties, attributed to its unique postsynaptic AMPA antagonism [32,38,39]. While it is not yet approved as a first-line treatment for focal epilepsy in many countries, this medication has potential and could be used at an early stage if our study confirms its effectiveness in prophylaxis against poststroke epilepsy.

The administration of AED or placebo for a period of 3 months is intended to intervene in the epileptogenesis phase, based on the relative duration of this phase observed in animal studies. Typically, the epileptogenesis phase in animal studies lasts for around 1 week, although this can vary depending on the animal model of seizure and epilepsy following brain injuries. By extrapolating from rat studies to human years [44], it is estimated that the epileptogenesis phase in rats is similar to around 3-4 months of human life. For the purposes of clinical feasibility, we administer a 3-month duration of AED.

In our trial to provide prophylactic treatment for epilepsy in patients who have not yet experienced seizures and are relatively vulnerable, particularly older patients who have endured stroke with multiple comorbidities, such as renal and liver diseases, we have opted for a relatively low medication dosage. Regarding the inclusion criteria, we do not enroll patients with severe renal or hepatic failure. However, we include patients who are already undergoing dialysis treatment. We assess the severity of liver disease using the Child-Pugh classification, with grade C being categorized as severe liver disease [45]. For the evaluation of renal disease severity, we use the National Kidney Foundation criteria, classifying stage 4 and above as severe renal disease [46]. In accordance with the renal dose adjustments for LEV [47], dosages vary depending on the level of renal impairment. For patients with mild renal impairment (glomerular filtration rate [GFR] 60-89 mL/min/1.73 m^2^), the recommended dosage range is 1000-2000 mg per day. For those with moderate renal impairment (GFR 30-59 mL/min/1.73 m^2^), the dose ranges from 500 to 1500 mg per day, while patients with severe renal impairment (GFR 15-29 mL/min/1.73 m^2^) are prescribed doses of 500-1000 mg per day. For individuals undergoing hemodialysis, the recommended dosage is 500-1000 mg per day with an additional 250-500 mg postdialysis supplement.

Our decision regarding medication dosages is guided by the World Health Organization’s Defined Daily Dose (DDD) guidelines [48] and our collective clinical experience. The DDD represents the estimated average daily maintenance dose for a drug when used for its primary indication in adults, taking into account our clinical expertise. For PER, the DDD is 8 mg, while for LEV, it is 1500 mg. In our study, we have chosen to administer PER at a dosage of 4 mg and LEV at 1000 mg. For LEV, this choice is driven by the fact that the majority of our recruited patients have normal renal function, with only a relatively small subset experiencing renal impairment (mild, moderate, or hemodialysis). To ensure randomization and maintain blinding in our triple-blind study, we have opted for a lower dose of 1000 mg per day. Of note, our blinding design excludes the use of postdialysis supplemental dosing.

According to the existing literature, late-onset seizures following a stroke typically exhibit a peak incidence between 6 and 12 months after the initial event [1]. Studies have shown that after an ischemic stroke, the highest risk for the occurrence of the first unprovoked poststroke seizure is observed during the initial follow-up year [5,49]. Furthermore, another study has reported the annual event risk of seizures after the first-ever ischemic stroke, indicating a risk of 6.3% after 1 year, 2.4% after 2 years, 1.3% after 3 years, and 0.3% thereafter [50]. Although the annual risk of seizures may indeed increase in subsequent years, given the highest risk observed during the first year and considering the practical feasibility of our protocol, we have chosen an 18-month follow-up period at this stage.

In terms of stroke severity, minor strokes (those with a small lesion size) are less likely to lead to the development of poststroke epilepsy. For each patient, we routinely arrange for MRI scans as a standard imaging procedure to assess the stroke, including its size, location, and the presence or absence of hemorrhagic transformation during admission. We do not typically perform brain computed tomography (CT) scans. It is worth noting that the absence of hemorrhagic transformation at the onset of the stroke does not eliminate the possibility of it occurring later during the acute stage. In the event of acute deterioration in neurological symptoms and signs, we arrange for follow-up brain MRI or CT scans and provide standard care.

In our protocol, we initiate treatment for patients with PLEDs. PLEDs have been considered to have a high association with seizures [51,52], and among the epileptiform abnormalities, the presence of lateralized periodic discharges was associated with a significantly increased risk of seizure [53]. In addition, PLEDs were associated with an acute process and occurred early during the course of the illness, with stroke being the main etiology [52].

While patients and research teams remain blinded to the allocated medications, the medication list, including cytochrome P450 inducers or inhibitors, is made available to study participants. This allows for vigilance regarding any potential drug-drug interactions. Moreover, given that our study involves a randomized controlled trial with blinding, which necessitates careful control over factors like color, size, and quantity of both the active drug and placebo tablets, the use of a powdered form is the most practical approach for conducting this trial. It is worth noting that the oral suspension form is only available for one of the medications, and furthermore, these oral suspension forms come in different colors. Additionally, the cost associated with preparing a placebo suspension in this context is prohibitively high. Regarding the issue of bioavailability, there has been no direct comparative study on bioavailability between the tablet and powdered forms. We do have clinical experience using the powdered form for patients who face challenges swallowing tablets or those reliant on nasogastric tubes. This practice is consistent with the medication package insert instructions. With their availability in multiple formulations in the future, these AEDs could serve as a potential drug for epilepsy prophylaxis during epileptogenesis.

### Conclusions

We anticipate that the intervention group will experience a lower incidence and severity of poststroke epilepsy than the control group. We will be able to document the potential prophylactic effect of LEV and PER in poststroke seizures and epilepsy in patients with MCA infarct and determine the antiepileptogenetic potential of LEV and PER in patients with major ischemic stroke.

## Abbreviations

AED: antiepileptic drug
AMPA: alpha-amino-3-hydroxy-5-methyl-4-isoxazolepropionic acid
CT: computed tomography
DDD: Defined Daily Dose
EEG: electroencephalogram
GABA: gamma-aminobutyric acid
GFR: glomerular filtration rate
IRB: institutional review board
LEV: levetiracetam
MCA: middle cerebral artery
MRI: magnetic resonance imaging
NIHSS: National Institutes of Health Stroke Scale
PER: perampanel
PLED: periodic lateralized epileptiform discharge

## Data Availability

The data sets generated and analyzed during this study are available from the corresponding author on reasonable request.
